# Morphometrical diagnosis of the malaria vectors *Anopheles cruzii, An. homunculus* and *An. bellator*

**DOI:** 10.1186/1756-3305-5-257

**Published:** 2012-11-13

**Authors:** Camila Lorenz, Tatiani Cristina Marques, Maria Anice Mureb Sallum, Lincoln Suesdek

**Affiliations:** 1 Programa de Pós-graduação (Mestrado) em Ciências (Biologia da Relação Patógeno-Hospedeiro), Instituto de Ciências Biomédicas, Universidade de São Paulo, 1500, 05509–300 São Paulo-SP, Brazil; 2 Departamento de Epidemiologia, Faculdade de Saúde Pública, Universidade de São Paulo, 1500, 05509–300 São Paulo-SP, Brazil; 3 Laboratório de Parasitologia, Instituto Butantan, Avenida Vital Brasil, 1500, 05509–300 São Paulo-SP, Brazil

**Keywords:** Culicidae, *Kerteszia*, Wing geometric morphometrics, Identification, Malaria vectors, Atlantic Forest

## Abstract

**Background:**

*Anopheles* (*Kerteszia*) *cruzii* is a primary vector of *Plasmodium* parasites in Brazil’s Atlantic Forest. Adult females of *An. cruzii* and *An. homunculus*, which is a secondary malaria vector, are morphologically similar and difficult to distinguish when using external morphological characteristics only. These two species may occur syntopically with *An*. *bellator*, which is also a potential vector of *Plasmodium* species and is morphologically similar to *An. cruzii* and *An. homunculus*. Identification of these species based on female specimens is often jeopardised by polymorphisms, overlapping morphological characteristics and damage caused to specimens during collection. Wing geometric morphometrics has been used to distinguish several insect species; however, this economical and powerful tool has not been applied to *Kerteszia* species. Our objective was to assess wing geometry to distinguish *An. cruzii*, *An. homunculus* and *An. bellator*.

**Methods:**

Specimens were collected in an area in the Serra do Mar hotspot biodiversity corridor of the Atlantic Forest biome (Cananeia municipality, State of Sao Paulo, Brazil). The right wings of females of *An. cruzii* (n= 40), *An. homunculus* (n= 50) and *An. bellator* (n= 27) were photographed. For each individual, 18 wing landmarks were subjected to standard geometric morphometrics. Discriminant analysis of Procrustean coordinates was performed to quantify wing shape variation.

**Results:**

Individuals clustered into three distinct groups according to species with a slight overlap between representatives of *An. cruzii* and *An. homunculus*. The Mahalanobis distance between *An. cruzii* and *An. homunculus* was consistently lower (3.50) than that between *An. cruzii* and *An. bellator* (4.58) or *An. homunculus* and *An. bellator* (4.32). Pairwise cross-validated reclassification showed that geometric morphometrics is an effective analytical method to distinguish between *An*. *bellator*, *An. cruzii* and *An. homunculus* with a reliability rate varying between 78-88%. Shape analysis revealed that the wings of *An. homunculus* are narrower than those of *An. cruzii* and that *An. bellator* is different from both of the congeneric species.

**Conclusion:**

It is possible to distinguish among the vectors *An. cruzii*, *An. homunculus* and *An. bellator* based on female wing characteristics.

## Background

The genus *Anopheles* of the family Culicidae contains species that are widely distributed throughout South America [1]. These species are commonly associated with watercourses and forests, frequently in coastal areas. In Brazil, the primary anopheline species involved in *Plasmodium* transmission belong to two subgenera, i.e., *Nyssorhynchus* (*Anopheles darlingi*, *An. aquasalis*, *An. nuneztovari* s.l., *An. oswaldoi*, *An. triannulatus* s.l. and species of the *An. albitarsis* complex) [2,3] and *Kerteszia* (*An. cruzii, An. bellator* and *An. homunculus*) [4], with *An. cruzii* and *An. darlingi* as the primary vectors of *Plasmodium* species [5]. The autochthonous transmission of extra-Amazonian malaria occurs mainly in areas of the Southeastern coastal Serra do Mar mountain range, where *An. cruzii* is a primary vector [6]. *Anopheles homunculus* and *An. bellator* are also important secondary vectors of human *Plasmodium* in that region [7,8]. In this region, *An*. *bellator*, *An*. *cruzii* and *An*. *homunculus* occur in sympatry and are somewhat morphologically similar. Most *Kerteszia* species use bromeliad phytothelmata as larval habitats, with the exception of *An. bambusicolus,* whose habitat is water accumulated inside bamboo internodes [9].

**Figure 1:**
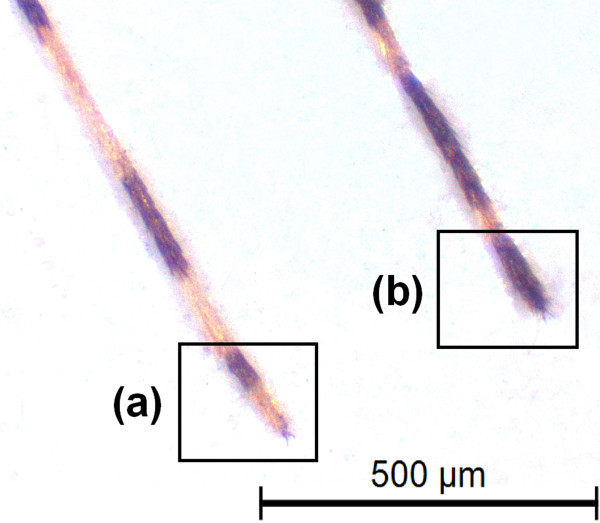
**Morphological differentiation of 5th posterior tarsomere visualized in adults of *An. cruzii *(a) and *An. bellator *(b).**

According to Harrison *et al.*[10], the females of *An. cruzii* and *An. homunculus* can be distinguished by the scaling on maxillary palpomeres 3 and 4. In addition, Martins [11] showed that the integument of the abdominal terga in *An. cruzii* females are uniformly reddish or reddish with lighter portions, whereas the abdominal integument of *An. homunculus* are blackish with whitish areas. For larval differentiation, Lima [12] observed that the saddles of segment X are lightly sclerotised in *An. cruzii,* with either yellowish or reddish integument. By contrast, the larvae of *An. homunculus* have strongly pigmented saddles of segment X, with dark brown to blackish integument [13]. *Anopheles bellator* can be distinguished from *An. cruzii* and *An. homunculus* by the narrow apical pale bands on hind tarsomeres 2–4 (Figure 1), which are 30% or less of the length of the tarsomeres, and the typically entirely dark colouration of hind tarsomere 5; by contrast, hind tarsomeres 2–5 are 50% basal black and 50% apical pale in *An. cruzii* and *An. homunculus*[10]. The morphological identification of these species is often hampered by variability in the diagnostic characteristics and by damage to insect body parts caused by the capture procedure [13].

Recently published literature shows an increasing tendency towards assessing medically important insect species using geometric morphometrics [14-18], an analytical tool that allows for multivariate statistical descriptions of biological structures [19]. In insects, the wings are the main target for morphometrics because of their two-dimensional form and homologous vein patterns. In the present study, we used geometric morphometrics as a complementary and low-cost tool to identify vector species of the subgenus *Kerteszia,* focusing on three species that occur in areas of the coastal Atlantic Forest.

## Methods

### Biological sampling

All of the specimens were collected in an area of the Atlantic Forest biome in the municipality of Cananeia, State of São Paulo, Brazil, more specifically in the neighborhood of the Aroeira district (24°53’06” S / 47°51’01” W). Voucher specimens of *An. cruzii* and *An. homunculus* were deposited in the Coleção Entomológica de Referência, Faculdade de Saúde Pública, Universidade de São Paulo (FSP-USP), Brazil. Mosquito larvae and pupae were taken from water that had accumulated in bromeliad tanks in 2009 and reared in the laboratory until adult emergence under standard conditions of temperature, food availability and container size. Adult females of *An. bellator* were captured in July and November 2011 with Shannon traps and preserved in plastic vials with silica gel. Species were identified based on colour pattern of larvae, according to Lima [12].

### Material preparation and data acquisition

**Figure 2:**
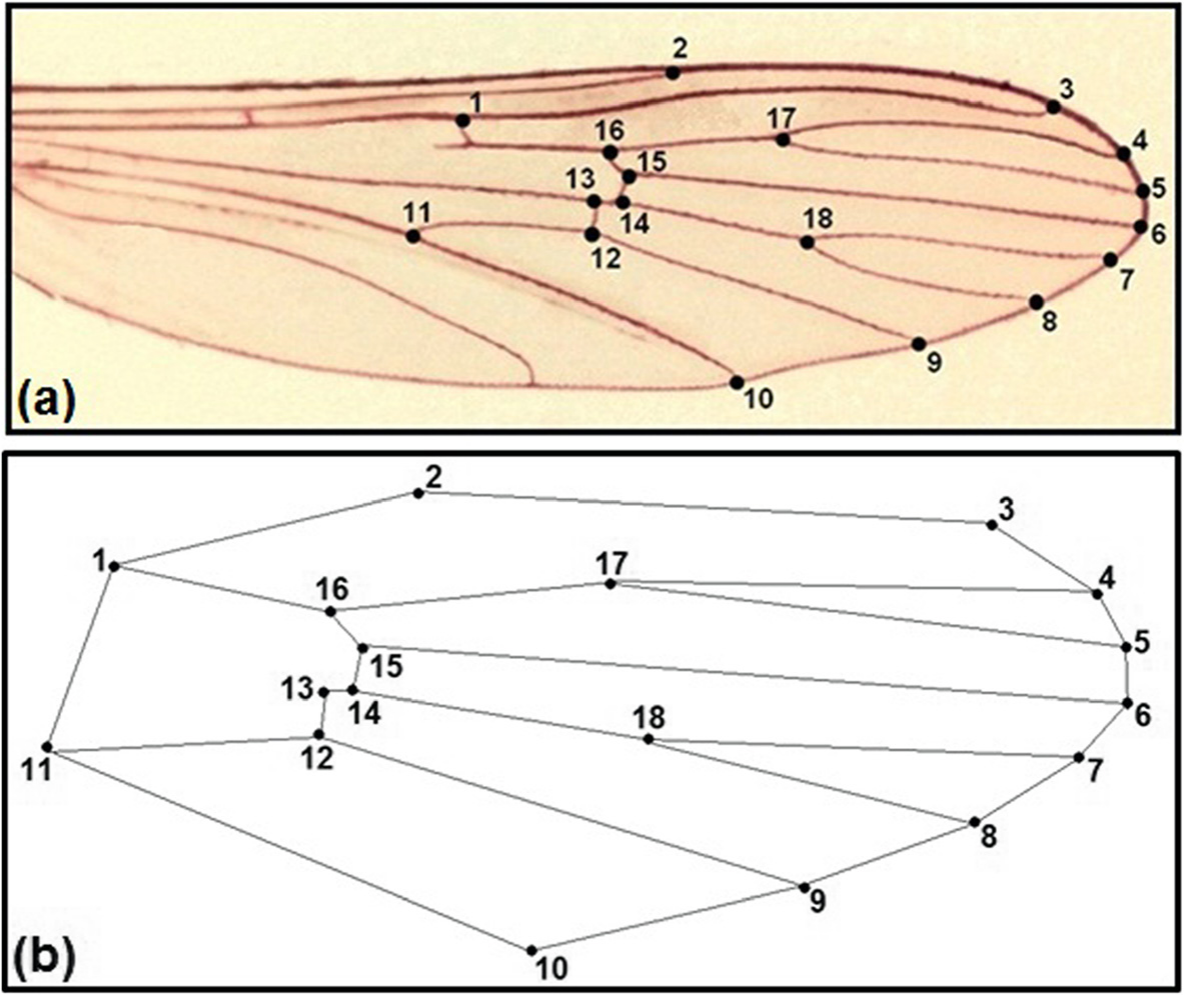
**(a) Wing of female *Anopheles bellator *colored with acid fuchsin showing the 18 landmarks selected for geometric morphometrics.** (**b)** Diagram of immaginary links between landmarks used to depict wing consensus.

The right wings of females of *An. cruzii* (n = 40), *An. homunculus* (n = 50) and *An. bellator* (n = 27) were removed from adults and cured before being mounted on microscope slides. The wings were soaked for 12 hours in a 10% potassium hydroxide (KOH) solution at room temperature to remove the wing scales. The potassium hydroxide was removed by washing the wings in a 20% solution of acetic acid. The wings were dyed with acid fuchsin for 60 minutes and then dehydrated in a series of ethanol concentrations ranging from 80% to 98%. Images of the wings were captured using a Leica DFC320 digital camera coupled to a Leica S6 microscope with 40X magnification. Eighteen wing landmarks (Figure 2) of each individual were digitised using the TpsDig software V.1.40 (QSC - James Rohlf), and the coordinate images were plotted onto a Cartesian plane for geometric descriptions. All of the wings were scanned twice, and the repeatability of the digitising procedure was assessed using statistical tests [20,21]. The wing pictures were deposited in the CLIC Image Bank (http://bioinfo-prod.mpl.ird.fr/morphometrics/clic/declic/list.html).

### Geometric morphometrics analysis

The wing size was estimated using isometric measurements of the centroid size [15,22]. Generalised Procrustes analysis was performed to assess the wing shape. To describe the shape variation without the effects of allometry, a regression analysis was performed between the coordinates of the landmarks and the centroid size for each of the three species.

Discriminant analysis of the canonical variables was performed. To test the accuracy of species classification yielded by morphometrics, each individual was reclassified by comparing the shape with the overall mean wing size of each species using the Mahalanobis distances. The reclassification was cross-validated and the distances were estimated in discriminant axes in the absence of the individual to be classified. Thin-plate splines were obtained by regression of the canonical scores *versus* the shape components. The morphometric statistical analyses were conducted with the software TpsUtil 1.29, TpsRelw 1.39 (QSC - James Rohlf), MorphoJ 1.02 and Statistica 7.0. The graphics were generated using Statistica and MorphoJ 1.02.

## Results

**Figure 3:**
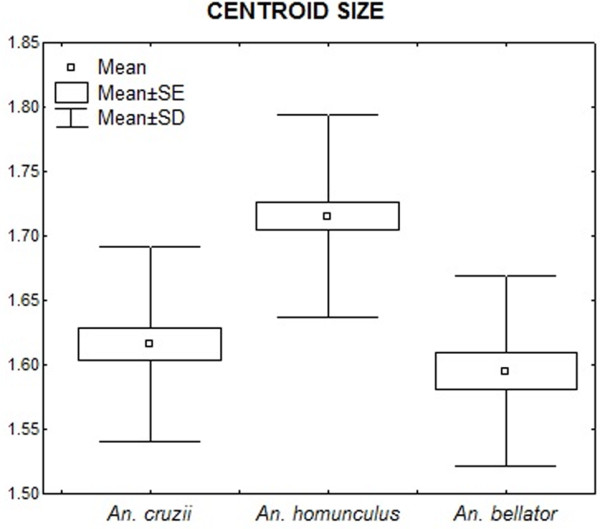
Descriptive statistics of centroid sizes (in mm).

The mean centroid size of *An. cruzii* was 1.62 mm (range 1.45 - 1.77 mm), that of *An. homunculus* was 1.71 mm (range 1.60 - 1.93 mm), and that of *An. bellator* was 1.59 mm (range 1.46 - 1.77 mm). Only the mean centroid size of *An. homunculus* (Figure 3) was significantly different from those obtained for the other two species (P<0.001; ANOVA + Tukey-Kramer post-hoc test). The allometric effect was low (5.47%) but statistically significant (P<0.0001) and was removed from the shape analyses. A second round of analyses were conducted, with the allometric effect included, however, the results were essentially similar.

**Figure 4:**
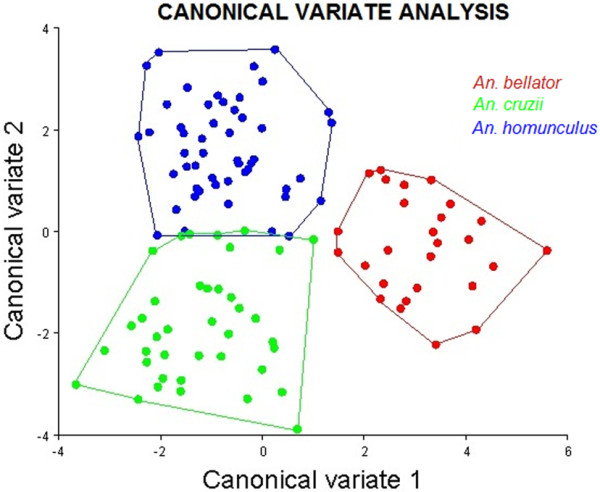
Morphological space of canonical variates resulting from comparison among the three species.

The canonical variate analysis for the wing shape showed that individuals clustered into distinct groups in the morphospace according to each species (Figure 4). *Anopheles bellator* was found to be isolated from the other species, whereas *An. cruzii* and *An. homunculus* slightly overlapped. The Procrustes distance between these two species was lower (3.50) than between *An. cruzii* and *An. bellator* (4.58) or between *An. homunculus* and *An. bellator* (4.32). The accuracy scores after a cross-validated reclassification test ranged from 78% to 88% (Table 1).

**Table 1:** Validated reclassification accuracies of each species derived from the three pairwise comparisons

**Compared species**	**Reclassification scores**
*An. cruzii*	82%
*An. homunculus*	82%
*An. cruzii*	82%
*An. bellator*	88%
*An. homunculus*	78%
*An. bellator*	84%

**Figure 5:**
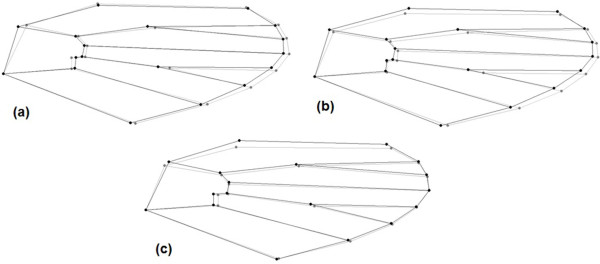
**Pairwise comparison of thin plate spline between species.** Landmarks displacement correspond to extreme of differentiation in each species. (**a**) *An. cruzii* (gray) and *An. bellator* (black); (**b**) *An. bellator* (black) and *An. homunculus* (gray) and (**c**) *An. homunculus* (gray) and *An. cruzii* (black).

Thin-plate splines with pairwise comparison of species evidenced higher displacement of certain landmarks that were more informative for species identification (Figure 5). The greatest distinction between *An. bellator* and *An. cruzii* was found with landmarks 1, 17 and 18, whereas the distinction between *An. bellator* and *An. homunculus* was most clear using landmarks 1, 2, 3, 17 and 18. The main shape differences when comparing *An. cruzii* and *An. homunculus* were observed in landmarks 1, 2, 3, 12 and 13.

**Figure 6:**
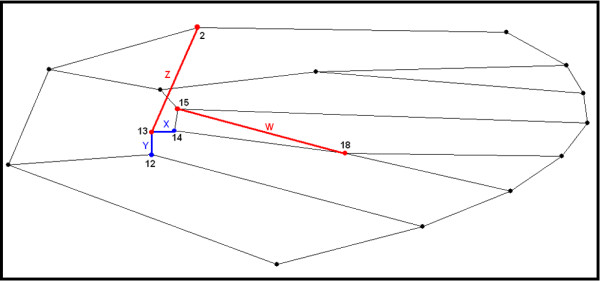
Graphical presentation of the distance between landmarks used for ratios x/y and z/w.

**Figure 7:**
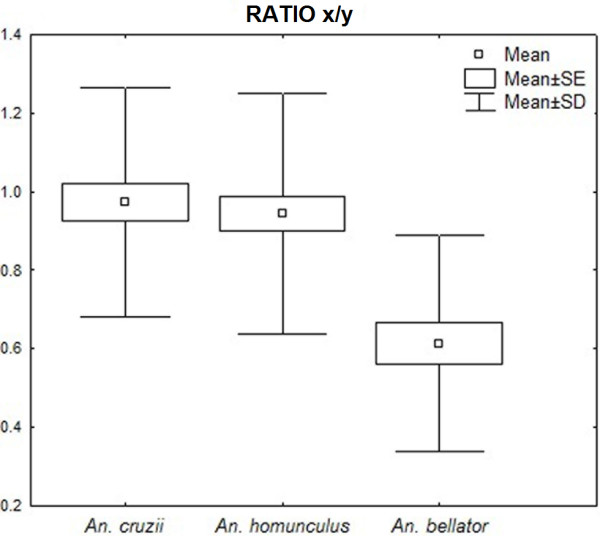
Descriptive statistics of ratio x/y (in mm).

**Figure 8:**
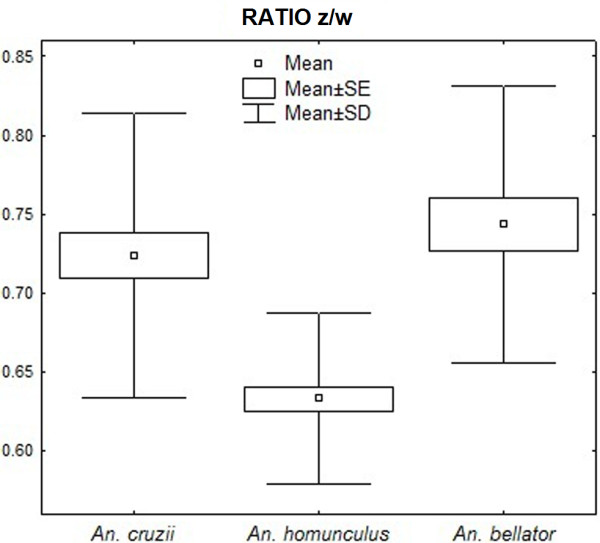
Descriptive statistics of ratio z/w (in mm).

We noted that distances between some most-influential landmarks were also conspicuously distinct between species: distance between landmarks 13–14 (distance x); 12–13 (distance y); 2–13 (distance z); and 15–18 (distance w). Ratios between those distances are depicted in Figure 6, which allow one to diagnose the species based on a single value. The mean ratio between dimensions x and y was 0.97 for *An. cruzii*, 0.94 for *An. homunculus* and 0.61 for *An. bellator* (Figure 7), with *An. bellator* being significantly different from the other species (T-test, P<0.0001). The z/w ratio was 0.72 for *An. cruzii*, 0.74 for *An. bellator* and 0.63 for *An. homunculus*, with *An. homunculus* being significantly different from the other species (T-test, P<0.0001) (Figure 8).

## Discussion

Adult females of *An. homunculus* and *An. cruzii* are morphologically similar; consequently, distinction between these species is generally problematic when using only the classical morphological characteristics described in identification keys [13]. The correct identification of species is essential for recognition of the vectors involved in the transmission of malaria and for helping researchers to develop control strategies [7]. Geometric morphometrics analyses revealed that *An. homunculus, An. cruzii* and *An. bellator* can be distinguished based on wing characteristics.

The results of the discriminant analysis showed that *An. bellator* is well separated from both *An. cruzii* and *An. homunculus* in the morphospace of the canonical variables. This finding is consistent with previous results indicating that A*n. bellator* can be easily distinguished by the adult external morphology (hindtarsomere 5). However, one would expect higher phenotypic similarity between *An. bellator* and *An. homunculus* if the wing shape is directly associated with the close phylogenetic relationships between the species [23]. *An. cruzii* and *An. homunculus*, which are occasionally misidentified [9], were also discriminated despite partial overlapping in the morphospace of canonical variables.

The wing shape divergence among these three species was not as significant as that for species of the genera *Culex* and *Aedes*[16,24], which may be a result of recent diversification of the subgenera [23] or due to evolutionary constraints. The close evolutionary relationships among *Kerteszia* representatives might be reflected in the wing shape because of the heritability of this structure, as proposed for other insects [12]. *Anopheles bellator* and *An. homunculus* can coexist in bromeliads and compete for resources [25], and this close association could impose constraints or favour canalisation to the observed phenotype.

Considering that the results obtained either with or without allometry were similar, we conclude that size variation did not interfere with species delimitation. *Anopheles homunculus* had the largest size among species in this study; however, we cannot ascertain that this comparison holds in nature because size is commonly subject to plasticity [15,24,26]. At least for *An. homunculus* and *An. cruzii*, it is plausible to consider that the size disparity may be associated with genetic determinism because both species were collected in the larval stage and reared to adults in the laboratory under similar environmental conditions and food resource availability.

Specimen collections were not simultaneous (years 2009 and 2011), what could lead us to believe that interspecific wing shape divergency may be partly a result of asynchronic sampling and microevolutionary changes. This idea is unlikely because the three-year interval is much shorter than the divergence time among the *Anopheles* species involved [23]. It has recently been reported that wing shape variation in *Aedes albopictus* can occur within four years [27] however, such variation is slight in comparison to macroevolutionary changes. Additionally, phenetic distance between *Ae. aegypti* and *Ae. albopictus* based on wing shape remained equivalent over several years [24].

In addition to helping taxonomists identify species, geometric morphometrics maybe used by health professionals to identify species in the future. This technique will not end up with other methods of identification, such as those based on the costal wing spots, but will complement them. Whereas present work was essentially based on wing landmarks, papers from Wilkerson and Peyton (1990) [28] and Motoki et al*.* (2009) [29] successfully used wing spot relative sizes to identify *Anopheles* species. Although it is not an easy task to simultaneously analyse landmarks and dark spots in *Anopheles* wings, we hope in a future to combine landmark and spot-based morphometrics, as suggested by Dujardin (personal communication: Jean Pierre Dujardin).

The mean ratio of dimensions x and y is 2/3 in *An. bellator,* whereas this ratio is nearly 1/1 in *An. cruzii* and *An. homunculus*. Remarkably, the length of segment 13–14 (distance x) in some individuals of *An. bellator* is so short that the segment is almost nonexistent. As far as we know, this vein pattern has not been observed in other culicids. Although the occasionally vestigial segment does not directly contribute to the diagnosis proposed here, it may be worth an investigation. As an example, the absence of a wing vein in *Drosophila melanogaster* was characterised as an informative mutation named *crossveinless*[30]. Additionally, the mean ratio z/w of *An. homunculus* was lower than those of the other two species. Accordingly, as shown in Figure 5, the wings of *Anopheles homunculus* are narrower anteroposteriorly.

Apart from the morphological characteristics used in this study, molecular taxonomic markers have been developed for *Kerteszia* species [13], facilitating species identification and delimitation. The employment of geometric morphometric methods in taxonomic studies is promising and should be performed in conjunction with other methods to facilitate the correct identification of anopheline species.

## Conclusion

The results of this study have provided data that will help in the correct identification of *Anopheles* species. It is possible to distinguish among the vectors *An. cruzii*, *An. homunculus* and *An. bellator* based only on female wing characters.

## Competing interests

The authors declare that they don’t have competing interests.

## Authors' contributions

CL, MAMS and LS conceived the study, carried out data analysis and results interpretation. TCM collected data in the field. CL and LS written the manuscript. All authors approved the final version of the manuscript.
